# Controlled human exposures to wood smoke: a synthesis of the evidence

**DOI:** 10.1186/s12989-020-00375-x

**Published:** 2020-10-02

**Authors:** Carley Schwartz, Anette Kocbach Bølling, Christopher Carlsten

**Affiliations:** 1 Department of Medicine, Division of Respiratory Medicine, University of British Columbia, P: 604-875-4729, 2775 Laurel Street 10th Floor, Vancouver, BC V5Z 1M9 Canada; 2 Section of Air Pollution and Noise, Department of Environmental Health, Norwegian Institute of Public Health, P.O. Box 222 Skøyen, 0213 Oslo, Norway

**Keywords:** Review, Wood smoke, Controlled human exposure, Airway, Systemic, Inflammation

## Abstract

**Background:**

Exposure to particulate matter (PM) from wood combustion represents a global health risk, encompassing diverse exposure sources; indoor exposures due to cooking in developing countries, ambient PM exposures from residential wood combustion in developed countries, and the predicted increasing number of wildfires due to global warming. Although physicochemical properties of the PM, as well as the exposure levels vary considerably between these sources, controlled human exposure studies may provide valuable insight to the harmful effects of wood smoke (WS) exposures in general. However, no previous review has focused specifically on controlled human exposure studies to WS.

**Results:**

The 22 publications identified, resulting from 12 controlled human studies, applied a range of combustion conditions, exposure levels and durations, and exercise components in their WS exposure. A range of airway, cardiovascular and systemic endpoints were assessed, including lung function and heart rate measures, inflammation and oxidative stress. However, the possibility for drawing general conclusions was precluded by the large variation in study design, resulting in differences in physicochemical properties of WS, effective dose, as well as included endpoints and time-points for analysis. Overall, there was most consistency in reported effects for airways, while oxidative stress, systemic inflammation and cardiovascular physiology did not show any clear patterns.

**Conclusion:**

Based on the reviewed controlled human exposure studies, conclusions regarding effects of acute WS exposure on human health are premature. Thus, more carefully conducted human studies are needed. Future studies should pay particular attention to the applied WS exposure, to assure that both exposure levels and PM properties reflect the research question.

## Background

Exposure to wood smoke (WS) is a global health risk. Despite the development of cleaner technologies for cooking and heating, too many people remain exposed to pollution from combustion of biomass, including wood. There are approximately 3 billion people who currently use cooking methods that produce indoor air pollution, whether through open fires or stoves, often fuelled by wood [1]. Indoor air pollution, including that due to wood combustion, presents a severe health risk to those exposed, causing nearly 4 million deaths globally each year [1]. Even in developed countries, wood stove usage for heating has increased, with approximately 1.9 million households in the US using wood as the main fuel for home heating in 2005, with an increase to up 2.5 million households in 2014 [2]. There is also a considerable contribution from residential wood combustion to outdoor air pollution, particularly in developed countries, that contributes significantly to human health risks [3].

Besides residential wood burning, sources of ambient biomass-derived exposure include wildfires and agricultural burning. In 2019, wildland fire accounted for approximately 29% of emissions of particulate matter of less than or equal to 2.5 μm in aerodynamic diameter (PM_2.5_) in the United States [4]. This contribution to air pollution is predicted to increase, as the frequency and intensity of wildfires are estimated to increase due to climate change [5]. It has been established through many studies that wildfire smoke exposure is linked to a host of adverse health effects [6].

Long-term inhalation of WS, in concentrations relevant for ambient exposure, induces mild inflammatory effects in the airways, systemic inflammation and decreased lung function in mice and rats [3]. Accordingly, epidemiological studies have associated WS exposure with increased mortality and morbidity, most notably linked to respiratory and cardiovascular conditions. In particular, associations between WS exposure and respiratory morbidity in terms of COPD and asthma, are strong. There are various proposed mechanisms to explain these effects (through cardiovascular, inflammatory and oxidative stress pathways) however these have yet to be confirmed [3, 6].

**Fig. 1:**
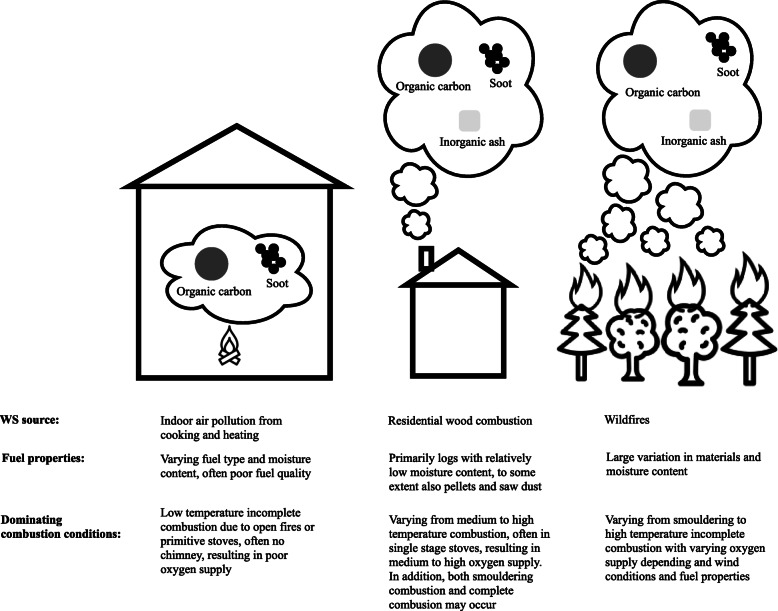
WS characteristics and sources

The physicochemical properties of WS particulate matter (PM) vary considerably according to the conditions under which they are generated (*eg*, contained stove, open fireplace, forest fire, etc). Emissions from residential wood stoves have been extensively characterized and include three main classes: i) ash particles emitted during complete combustion conditions (high temperatures and sufficient oxygen supply), ii) soot agglomerates originating from high temperature but air-starved combustion, and iii) organic-dominated emissions resulting from combustion at lower temperatures [3, 7]. Residential WS is likely to be dominated by one of these classes or contain significant fractions of several classes. Emissions from wildfires are not as well characterized [8] but are likely to contain a mixture of these classes, due to the mixed fuel and conditions occurring in such uncontrolled fires. A visualization of these classes of WS and their sources is included in Fig. 1.

The physicochemical properties of PM from WS define their intrinsic toxicity, but also their deposition probability and clearance rate. Whereas deposition of ash particles and organics has been reported to be low due to hygroscopic growth with calculated deposited fraction of 21–25% [9, 10], wood smoke particles from mixed wood combustion conditions appears to be higher (38%) probably due to presence of more hydrophobic particles [11]. Since the WS PM classes also differ in solubility, soot is insoluble while ash and organics are generally water soluble, the particle class also affects the clearance rate. Thus, the physicochemical properties of wood smoke PM are detrimental for their impact on human health [3].

Accordingly, in controlled human exposure studies, the conditions applied to generate WS are crucial, as they determine the physicochemical properties of the generated PM and thereby their potential to cause human health effects. Consequently, the choice of combustion conditions will result in data with particular relevance for different human exposure scenarios, such as residential combustion in developed countries, indoor air pollution in developing countries or wildfires.

Several reviews have been published looking at the health effects of WS exposure [3, 6, 12–15]. These have focused mainly on epidemiology, animal exposure, or in vitro studies. No previous review has focused specifically on controlled human exposure studies to WS. Therefore, the purpose of this review is to describe and summarize this particular group of studies, given their unique ability to provide detailed insight within the human context, in a manner that minimizes the risk for confounding. In doing so, we will attempt to distil patterns that emerge, elucidate gaps in knowledge and provide guidance for future directions of inquiry.

## Methods

**Fig. 2:**
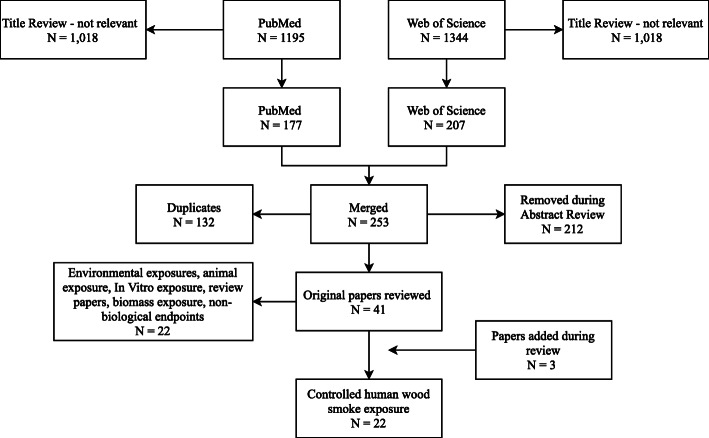
Review process algorithm

A PubMed and Web of Science search was performed to identify studies concerning controlled human exposures to WS. Controlled exposure was here defined as an experimental exposure where levels of PM and combustion gas exposure is pre-determined and maintained by the researchers (environmental exposure is eliminated). The search was limited to only include experiments involving human subjects. The search terms used were ((Wildfire) OR (Woodsmoke) OR ((Wood) AND (Smoke)) AND ((Human) (AND Exposure)) OR (Exposure)). This search strategy produced 1195 publications from PubMed and 1344 from Web of Science (August 2019). The number of publications were narrowed down to 177 from PubMed and 207 from Web of Science through title review. Between these, 132 were duplicates and another 212 were removed during the review of the publication abstracts. From these 41 remaining publications those that involved environmental (non-controlled) exposure, animal studies, in vitro studies, exposures other than WS or pellets, and other review publications were eliminated. This resulted in 22 publications that involved controlled human exposure to WS, with an additional 3 publications identified during review, resulting in a total of 22 (Fig. 2).

The exposure conditions applied in the 12 human exposure studies are grouped in terms of the PM class dominating the exposure (Table 1; organic carbon (OC), soot, inorganic ash, or a combination of these classes). The basis for the categorization is provided in Supplementary Material. For most American studies, the physicochemical properties of the PM applied is characterized based on provided information regarding stove type and fuel, including loading frequency, weight and humidity, in combination with available literature regarding WS PM characteristics (Supplementary material, Table S1).

**Table 1:** Exposure Conditions. The table displays a summary of the exposure conditions for each controlled human exposure study. Studies have been ordered by combustion conditions and PM emission class. For major endpoint of each publication: LF = Effects on Lung Function, AI = Airway Inflammation Effects, CCP = Effects on Circulating Cells and Proteins, OS = Effects on Oxidative Stress, CVP = Effects on Cardiovascular Physiology. All other abbreviations are denoted in the list of abbreviations in main text

Publications	Major end-point	Stove and fuel type	Wood species (Moisture)	Major PM class^a^	Study Design (Number of Participants)	Exposure length	Exercise	Exposure Conditions (PM_**2,5**_ in μg/m^**3**^) ^b^
**Ghio et al** [16]	LF, AI, CCP, OS, CVP	Electric element /logs	Red oak wood (−)	Organics	Crossover, sequential (10)	2 h	15 mins × 4 on bike	485
**Burbank et al** [17]	AI	Electric element / logs	Red oak (−)	Organics	No FA control (35)	2 h	15 mins × 4 on bike	500
**Rebuli et al** [18]	AI	Electric element / logs	Red oak (−)	Organics	Parallel, randomized (39)	2 h	None	488
**Fedak et al** [19]	CVP	Wood chips/wood sticks	Unknown	Organics/ soot	Crossover, randomized (48)	2 h	None	Gasifier – 46 Fan rocket – 95 Rocket elbow – 254 Three stone fire – 463
**Sehlstedt et al** [20]	LF, AI, OS	Pellet stove / pellet or sawdust	Softwood or mixture from pine and spruce (18%)	Organics/ soot	Crossover, randomized (19)	3 h	15 mins × 6 on bike	224
**Pope et al** [21]	CVP	Conventional/logs	Pine (−)	Organics/ soot	Crossover, randomized (14)	3 h	None	180
**Ferguson et al** [22]	AI, CCP	Conventional / logs	Western larch (15%)	Organics/ soot	Crossover, randomized (10)	1.5 h	Contin-uous on treadmill	Low – 253.9 High - 506.2
**Ferguson et al** [23]	LF							
**Peters et al** [24]	OS							
**Barregard et al** [25]	CCP, OS	Conventional / logs	50/50 mixture of birch/spruce (15–18%)	Organics/ soot	Crossover, sequential (13)	4 h	25mins × 2 on bike	243–279
**Barregard et al** [26]	AI, CCP, OS							
**Danielsen et al** [27] **Murgia et al** [28]	OS OS							
**Stockfelt et al** [29]	AI, CCP	Conventional / logs	50/50 mixture of birch/spruce (15–18%)	Organics/ soot	Crossover, sequential (16)	3 h	None	SUP - 221 BOP − 148
**Stockfelt et al** [30]	CCP, OS							
**Riddervold et al** [38]		Questionnaire						
**Riddervold et al** [31]	LF, AI	2-stage / logs	1 kg beech wood logs (16–20%)	Soot / ash	Crossover, randomized (20)	3 h	None	Low – 220.5 High – 354.38
**Forchhammer et al** [32]	CCP, OS, CVP							
**Bønløkke et al** [33]	CCP, CVP							
**Unosson et al** [34]	CVP	Conventional / logs	Birch (16–18%)	Soot / ash	Crossover, randomized (14)	3 h	15 mins × 6 on bike	314
**Muala et al** [35]	LF, AI, CCP, OS							
**Hunter et al** [36]	CCP, CVP	Conventional / logs	Birch (16–18%)	Soot / ash	Crossover, randomized (16)	1 h	15 mins × 2 on bike	1115

^a^See Table S1 for more details regarding PM class

^b^ PM_2.5_ for filtered air control ranged between 3 and 18 μg/m^3^ (excluding Burbank et al [17] that did not include a filtered air control)

## Results

### Summary of study characteristics

The 23 identified publications were based on 12 human controlled exposure studies. The duration of the exposure varied from 1 to 4 h and seven of the studies included an exercise component (Table 1). The PM mass concentrations applied varied among the studies, ranging from ~ 100 to 1000 μg/m^3^. Studies varied in size and design with the number of subject ranging from 10 to 48 participants. The majority (7) of the studies use a randomized double-blinded crossover exposure design with 3 studies non-randomized and 2 studies non-crossover. The degree of physical activity also varied between the studies, affecting the PM deposition and therefore the effective dose, as exercise can increase particle deposition by several fold [37]. Thus, in studies including an exercise component, the effective dose is likely to be considerably higher than in studies with similar PM concentration and exposure duration but without an exercise component. Since the physicochemical properties of the applied WS PM also affects the effective dose, the exposure concentrations indicated in Table 1 should be interpreted with caution.

The stove, fuel type, and combustion conditions also varied among the studies, conferring differences in the physicochemical properties of the applied WS PM. Since these properties affect both the intrinsic PM toxicity as well as PM deposition probability and clearance rate, this information is of importance in the comparison and interpretation of studies. The studies performed in the Nordic countries generally provide a thorough characterization of the physicochemical properties of the WS, such as chemical analysis of polycyclic aromatic hydrocarbons (PAHs) and alkali metals, particle numbers, content of elemental and organic carbon (EC and OC, respectively) etc. In contrast, other studies generally limit their exposure characterization to the PM mass and number concentrations and, in some cases, associated gasses. For the purpose of this review, publications have been sorted by WS emission classes/combustion conditions from incomplete combustion to high temperature complete combustion in all tables.

For smoldering combustion (air-starved combustion in a conventional wood stove without advanced combustion technology), the emissions are dominated by organics [16–19]. Similarly, air-starved operation of a pellet stove [20] will also result in emissions dominated by organics, although soot and ash particles are also emitted. With increasing temperatures and air supply, soot aggregates will be formed, resulting in emission of a mixture of soot and organics from flaming combustion in stoves, fireplaces and open fires [21–30]. Upon even more complete combustion conditions (*eg*, highest temperatures in stoves with good air supply) soot is reduced to inorganic ash, consisting of elements that are refractory to combustion [31–33, 38]. Emissions from optimal operation of pellet stoves is dominated by these water-soluble ash particles. High temperature combustion in conventional wood stoves with insufficient air supply will result in emissions dominated by soot containing high PAH levels and, depending on the air supply, formation of inorganic ash may also take place [19, 34–36]. The dominating PM class for each study is listed in Tables 1,2,3,4,5, and studies are sorted by combustion conditions and/or dominating PM class.

**Table 2:** Effects of WS exposure on airway inflammation. The table displays the significant effects of WS exposure per publication or effects that were reported in multiple publications (biomarkers only reported in one publication with non-significant results not included). A blank space indicates that the endpoint was not included in that study, ns indicates no significant effect observed, while arrows indicate significant increase (↑) or decrease (↓) due to WS exposure. All time points are taken with 0 h = start of controlled exposure. Abbreviations are denoted in the list of abbreviations in main text

	Ghio et al [16]	Burbank et al [17]	Rebuli et al [18]	Sehlstedt et al [20]	Ferguson et al [22]	Barregard et al [25]	Stockfelt et al [29]	Riddervold et al [31]	Muala et al [35]
**Exposure**									
Major PM class	OC	OC	OC	OV/Soot	OC/Soot	OC/Soot	OC/Soot	Soot/Ash	Soot/Ash
Exposure time	2 h	2 h	2 h	3 h	1.5 h	4 h	3 h	3 h	3 h
Exercise (y/n)	yes	yes	no	yes	yes	yes	no	no	yes
PM_2.5_ conc. (μg/m^3^)	485	500	488	224	low – 253.9 high – 506.2	243–279	SUP – 221 BOP – 148	low – 220.5 high – 354.3	314
**Biomarkers**									
**FeNO**									
FeNO_50_				ns		ns	↑ at 24 h (BOP)	ns	ns
FeNO_270_				ns		↑ at 7 h	↑ at 24 h, 48 h (SUP) ↑ at 8 h, 24 h, 48 h (BOP)	ns	
**Alveolar NO**						↑ at 7 h			
**EBC pH**					↓ 1.5 h (high)			↑ at 6 h	
** BAL cell count**									
Neutrophil	↑ at 20 h			ns					ns
Lymphocyte				ns					↓ at 24 h
** BAL cytokines**									
IL-6	ns								ns
MMP-9				ns					ns
MPO				ns					ns
** BW cell counts**									
Neutrophil	↑ at 20 h			ns					↓ at 24 h
** BW cytokines**									
IL-6	ns								ns
MMP-9				ns					↓ at 24 h
MPO				ns					↓ at 24 h
sICAM1									↓ at 24 h
LDH									↓ at 24 h
** Bronchoscopy biopsy cell counts**									
Submucosal									CD3+ lymphocytes at ↑ 24 h Mast Cells at ↑ 24 h
Epithelial									CD3+ lymphocytes at ↑ 24 h CD8+ lymphocytes at ↑ 24 h
**NAL cytokines**									
IP-10			↓ at 48 h						
IL-6			↓ at 48 h					ns	
**NAL gene expression**			Sex-specific Changes						
**Sputum %PMNs**		↑ at 24 h

**Table 3:** Effects of WS exposure on circulating cells and proteins. The table displays the significant effects of WS exposure per publication or effects that were reported in multiple publications (biomarkers only reported in one publication with non-significant results not included). A blank space indicates that the endpoint was not included in that study, ns indicates no significant effect observed, while arrows indicate significant increase (↑) or decrease (↓) due to WS exposure. All time points are taken with 0 h = start of controlled exposure. Abbreviations are denoted in the list of abbreviations in main text

	Ghio et al [16]	Ferguson et al [22]	Barregard et al [25, 26]	Stockfelt et al [29, 30]	Forchhammer et al [32] Bønløkke et al [33]	Muala et al [35]	Hunter et al [36]
**Exposure**							
Major PM class	OC	OC/Soot	OC/Soot	OC/Soot	Soot/Ash	Soot/Ash	Soot/Ash
Exposure time	2 h	1.5 h	4 h	3 h	3 h	3 h	1 h
Exercise (y/n)	yes	yes	yes	no	no	yes	yes
PM_2.5_ conc. (μg/m^3^)	485	224	243–279	SUP- 221 BOP – 148	low – 220.5 high – 354.38	314	1115
**Biomarkers**							
**Serum haemoglobin**	ns		ns [25]				
**Serum haematocrit**	ns		ns [25]				
**Serum cell counts**							
Platelets	ns		ns [25]	↓ at 7 h (BOP), 24 h (SUP/BOP) [30]			ns
Neutrophils	↑ at 20 h						ns
Lymphocytes						various subsets ↑ at 27 h	ns
Leukocytes	ns		ns [25]	ns [30]			ns
RBC	ns		ns [25]				
**Serum proteins**							
CRP			ns [25] [26]	↓ at 7 h (SUP) [30]			
SP-A				ns [29]	ns [33]		
SP-D		ns		↓ at 7 h (BOP) [29]	ns [33]		
SAA			↑ at 4 h, 7 h, and 24 h [25] ns [26]	ns [30]			
Fibrogen			ns [26]	↓ at 7 h (BOP), 24 h (SUP/BOP) [30]			
Factor VII			ns [25]	↑ at 7 h, 48 h (BOP) [30]			
vWf	ns		ns [25]	ns [30]	ns [33]		
**Serum D-dimers**	ns		ns [25]	↓ at 48 h (SUP) [30]			
**Serum soluble adhesion molecules**							
sP-selectin				ns [30]	ns [33]		
sVCAM-1				↓ at 7 h (SUP/BOP) [30]			
sICAM-1				↑ at 44 h (BOP) [30]	ICAM-1 ns [32]	ns	
**Serum cytokines**							
IL-6	ns		↓ at 7 h [25]	ns [30]	↓ at 9 h [33]	ns	
TNF- α	ns		ns [25]		ns [33]	ns	
IL-1β	↑ at 20 h				↑ at 3 h [33]		
**Serum COHb**	ns						↑ at 3 h
**Serum CC16**			↑ at 24 h [26]	↑ at 7 h (SUP) [29]	ns [33]	ns	
**Urine CC16**			ns [26]	↑ at 7 h (SUP) [29]	ns [33]

**Table 4:** Effects of WS exposure on markers of oxidative stress. The table displays the significant effects of WS exposure per publication or effects that were reported in multiple publications (biomarkers only reported in one publication with non-significant results not included). A blank space indicates that the endpoint was not included in that study, ns indicates no significant effect observed, while arrows indicate significant increase (↑) or decrease (↓) due to WS exposure. All time points are taken with 0 h = start of controlled exposure. Abbreviations are denoted in the list of abbreviations in main text

	Ghio et al [16]	Sehlstedt et al [20]	Ferguson et al [22] Peters et al [24]	Barregard et al [25, 26] Danielsen et al [27] Murgia et al [28]	Stockfelt et al [29, 30]	Forchhammer et al [32]	Muala et al [35]
**Emissions**							
Major PM class	OC	OC/Soot	OC/Soot	OC/Soot	OC/Soot	Soot/Ash	Soot/Ash
Exposure time	2 h	3 h	1.5 h	4 h	3 h	3 h	3 h
Exercise (y/n)	yes	yes	yes	yes	no	no	yes
PM_2.5_ conc. (μg/m^3^**)**	485	224	low – 253.9 high – 506.2	243–279	SUP – 221 BOP – 148	low – 220.5 high – 352.38	314
**Biomarkers**							
**Serum UA**^**a**^			↓ at 1.5 h (combined) [24]				
**Serum TEAC**^**a**^			↑ at 1.5 h, 2.5 h (combined) [24]				
**Serum oxidative damage markers**							
8-iso			↑ at 1.5 h (high and low) [24]				
LOOH			↓ at 2.5 h (combined) [24]				
3-NT			↑ at 1.5 h (combined) [24]				
**Serum MPO**			↓ at 1.5 h [24]				
**Serum modified purines**							
hOGG1				↑ at 24 h [27]			
oGG1						ns	
**Serum FPG sites**				ns [27]		ns	
**Urine 8-iso-PGFa**			↑ at 24 h [22]		↓ at 24 h (BOP), 44 h (SUP/BOP) [30]		
**EBC oxidative damage markers**							
8-iso			↑ at 2.5 h (combined) [22]	ns [28]			
MDA				↑ at 24 h [26]	ns [30]		
**BAL markers**							
MPO		ns					ns
GSH		↑ at 24 h					ns
GSx							↑ at 24 h
GSSG		ns					ns
**BW markers**							
MPO		ns					↓ at 24 h
GSH		ns					ns
GSSG		ns					ns

^**a**^UA and TEAC are considered indicators of oxidative stress. See [39, 40] for more details

**Table 5:** Effects of WS Exposure on Cardiovascular Physiology The table displays the significant effects of WS exposure per publication or effects that were reported in multiple publications (biomarkers only reported in one publication with non-significant results not included). A blank space indicates that the endpoint was not included in that study, ns indicates no significant effect observed, while arrows indicate significant increase (↑) or decrease (↓) due to WS exposure. All time points are taken with 0 h = start of controlled exposure. Abbreviations are denoted in the list of abbreviations in main text

	Ghio et al [16]	Fedak et al [19]	Pope et al [21]	Forchhammer et al [32]	Bønløkke et al [33]	Unosson et al [34]	Hunter et al [36]
**Emissions**							
Major PM class	OC	OC/Soot	OC/Soot	Soot/Ash	Soot/Ash	Soot/Ash	Soot/Ash
Exposure time	2 h	2 h	3 h	3 h	3 h	3 h	1 h
Exercise (y/n)	yes	no	no	no	no	yes	yes
PM_2.5_ conc. (μg/m^3^**)**	485	Gasifier – 46 Fan rocket – 95 Rocket elbow – 254 Three stone fire – 463	148	low – 220.5 high – 352.38	low – 220.5 high – 354.38	314	1115
**Biomarkers**							
**HRV**							
SDNN, RMSSD, pNN50	ns				ns	↓ at 3 h	
Normalized High Frequency	ns				ns	↑ at 3 h	
High/Low Frequency	ns				ns	ns	
**Heart rate**	maximal - ↓ at 2 h				ns	↓ at 3 h	ns
**Blood pressure**		↓ systolic pressure at 3 h (three stone fire) ↑ systolic pressure at 24 (gasifier, fan rocket, three stone fire)				ns	ns
**MVF/MVRI**			ns	ns			
**Arterial stiffness**						↑ at 3 h	ns
**Forearm blood flow - bradykinin infusion**							↑ at 1 h
**ECG**	ns				ns	Augmentation Index - ↑ at 3 h Augmentation Pressure - ↑ at 3 h Pulse Wave Velocity - ↑ at 3 h	ns

Four main groups of biological endpoints were assessed in the publications: airway inflammation, systemic effects, markers of oxidative stress, and changes in cardiovascular physiology (Tables 2,3,4,5). The following will summarize each of the publications resulting from studies that contributed to the collection of endpoint data with regard to WS exposures.  One study only assessed subjective symptoms by a questionnaire and is not included in the following [38].

### Study-specific methods and outcomes

Ghio et al [16] investigated the effect of WS generated by smouldering combustion conditions on pulmonary and systemic inflammation. Participants were exposed for 2 h to organics-dominated red oak WS created through an electric heating element placed inside a conventional woodstove. Deposition of the WS was also increased through 15-min intervals of exercise and rest on a stationary bike. Neutrophils in blood increased significantly immediately after exposure. In addition, the neutrophil numbers increased significantly in blood, bronchoalveolar lavage (BAL; a more distal airway sampling technique) and bronchial wash (BW; a more proximal airway sampling technique) samples 20 h after exposure. The pro-inflammatory cytokine interleukin 1 beta (IL-1β) and the toxicity marker lactate dehydrogenase (LDH) also increased significantly in blood after WS exposure. This publication also found changes in cardiovascular physiology with a significant decrease in maximal heart rate immediately following WS exposure. Although this is the first controlled human WS exposure study to show an increased recruitment of neutrophils in the lungs, the concentrations applied were in the high range (500 μg/m^3^).

Similar WS exposure conditions to those described in Ghio et al [16] were also used by Burbank et al [17] and Rebuli et al [18], although the latter did not include the exercise component. Burbank et al [17] reported significantly increased percentages of neutrophils in sputum 24 h after WS exposure. The impact of subjects’ glutathione S-transferases M1 (GSTM1) genotype was also assessed, and WS exposure induced a greater change from baseline in the GSTM1-null subjects than GSTM1-sufficient participants. These results add to the evidence that WS exposure may induce an inflammatory response in the airways, and also suggest that individuals with GSTM1-null genotype may be more susceptible to these effects.

Rebuli et al [18] followed exposure to either filtered air (FA) or WS with administration of live attenuated influenza virus to determine how WS exposure would affect the immune response to the virus. Of all the cytokines measured in nasal lavage (NAL) samples, only interferon gamma-induced protein 10 (IP-10) levels were affected (decreased levels 48 h after WS exposure). This publication also noted sex-specific changes attributable to WS in gene expression of 13 genes in males and the downregulation of 18 genes in female participants.

Fedak et al [19] investigated effects of a variety of WS sources on blood pressure. This publication specifically investigated small stove technologies using stove-specific exposure concentrations based on expected real-life exposures. For this review, only stoves using wood as a fuel source are included, i.e. gasifier, fan rocket, rocket elbow and three stone fire, which resulted in EC or OC dominated exposures. Participants were exposed to WS or FA for 2 h with a 2-week washout period between exposures. Brachial blood pressure was taken at baseline, immediately post, 3 h-post, and 24 h-post exposure. The results from this study showed that there was a significant increase in systolic pressure 24 h following all WS exposures (excluding the rocket elbow stove condition). Despite application of different WS PM concentrations, no dose response was noted in the results.

Sehlstedt et al [20] utilized a pellet stove with a pine and spruce mixture to create organics-dominated WS (with soot and inorganic ash). Participants were exposed for 3 h with 15 min intervals of rest and exercise on a stationary bike. This publication investigated the effects of WS exposure on airway inflammation and lung function. The glutathione (GSH) levels were significantly increased in BAL 24 h after exposure, while no other markers of inflammation or antioxidant defence were significantly affected in the airways due to the WS exposure.

Pope et al [21] used an exposure setup that enabled investigation of UV-aged WS on vascular function. WS was generated in a conventional stove which was then treated with a catalyst to convert CO to CO_2_ followed by UV light treatment to artificially age the WS to more closely resemble atmospheric conditions. The exposures were dominated by OC or soot (Supplementary material, Table 1). Participants were exposed to WS for 3 h followed by 3 h exposure to FA. After a week wash-out period this protocol was repeated with the exposure conditions reversed. The microvascular responsiveness index (MVRI) was measured for each participant at baseline and immediately after each 3-h exposure. The results from this study showed no significant changes in MVRI due to WS exposure.

Effects of WS exposure on both airway and systemic inflammatory markers were assessed by Ferguson et al [22]. Here a conventional woodstove burned Western larch for 1.5 h to generate soot/organics-dominated WS while participants continually exercised on a treadmill. From this, pentraxin-3 in the blood was significantly increased immediately and 1-h post WS exposure. In exhaled breath condensate (EBC) samples, 8-isoprostane (8-iso) increased 1 h post-exposure and the pH of the sample decreased immediately post-exposure. There were also some observed changes in myeloperoxidase (MPO) and pentraxin-3 in EBC but these results did not reach significance. The authors concluded that these results suggest that there were some trends to show that this type of exposure leads to airway and systemic inflammatory effects.

Ferguson et al [23] reported effects on lung function from the same human controlled exposure study. Although a slight decrease in forced expiratory volume in 1 s (FEV_1_), forced vital capacity (FVC), and the ratio of these values (FEV_1_/FVC) was observed, none of these results reached significance. With regard to oxidative stress markers in the same study, Peters et al [24] reported that uric acid (UA) values decrease immediately post-exposure and plasma Trolox equivalent antioxidant capacity (TEAC) levels were increased both immediately after and 1 h after the exposure. A biomarker for plasma oxidative stress, lipid hydroperoxides (LOOHs), was decreased 1 h post-exposure, while 8-iso, 3-nitrotyrosine (3-NT) and plasma MPO increased immediately post WS-exposure. The authors concluded that these results suggest that WS exposure may cause/contribute to systemic oxidative stress.

For Barregard et al [25, 26] Danielsen et al [27] and Murgia et al [28], a conventional cast iron woodstove burned a mixture of birch and spruce logs to produce soot/organics-dominated WS. Participants were exposed for 4 h with two 25-min intervals of exercise on a stationary bike. In Barregard et al [25], the effect of WS exposure on cardiovascular endpoints was assessed through biomarkers of inflammation, coagulation, and oxidative stress. There was a significant increase in serum Amyloid A (SAA) and the ratio of factor VIII complex and the von Willebrand factor (VIIIc/vWf) at both 3 h and 20 h post exposure. Moreover, at 20 h post-exposure the factor VIIIc increased in the blood and in 8-iso-prostaglandin F2 alpha (8-iso-PGF2α) in the urine. The authors interpret these observations as evidence for increased vascular and systemic inflammation from the WS exposure that may increase the risk of cardiovascular adverse outcomes.

With regard to pulmonary effects in the same study, Barregard et al [26] reported effects of the WS exposure on markers of pulmonary inflammation and oxidative stress. Malondialdehyde (MDA) in breath condensate increased immediately post-exposure and 20 h post WS exposure. Exhaled nitric oxide (NO) was also shown to increase at 3 h post-exposure. There was also a net increase in serum club cell protein-16 (CC16) 20 h after WS exposure. These findings suggest that exposure to WS could cause distal pulmonary inflammation and oxidative stress.

Danielsen et al [27] assessed markers of oxidative DNA damage due to the WS exposure. A marker for DNA repair activity, hOGG1 mRNA, was significantly increased in blood due to the exposure, while the number of DNA strand breaks was decreased. In contrast, other markers of oxidative stress, including urinary excretion of 8-oxo-2′-dexyguanosine (8-oxodG) and 8-oxo − 7, 8-dihydroguanine (8-oxoGua), mRNA expression levels of human MutT homolog (hNUDT1) and histone H1 (hHO1), and human 8-oxoguanine glycosylase (hOGG1) activity level, were not affected by WS exposure. These results suggest that there was detectable upregulation of DNA damage repair mechanisms but no detectable direct genotoxic effect was found.

The final publication from this study [28] investigated the effects of WS exposure on oxidative stress through the changes of markers in EBC and urine. Murgia and colleagues reported no significant net changes in 8-iso in EBC, however there were significant inverse correlations with other biomarkers of oxidative stress and inflammation from previous publications [26].

In Stockfelt et al [29, 30] a similar setup was used to Barregard at al [25, 26]. except that WS exposure of two different phases of the combustion cycle were used, namely the start-up phase (SUP) and burn-out phase (BOP) creating soot/organics-dominated WS. For each exposure type (FA, SUP, and BOP) participants were exposed for 3 h at rest. Stockfelt et al [29] reported an increase in CC16 in the blood, 7 h post-exposure start and 24 h post-exposure start in the urine from the SUP exposure. There was also a decrease in surfactant protein D (SP-D) in the blood 7 h post-exposure start to BOP. Finally, the levels of fractional exhaled NO (FeNO50 and FeNO270) increased significantly post exposure (see Table 2 for more details). The authors concluded that these results showed a link between WS exposure and inflammatory response in the airway.

Stockfelt et al [30] assessed markers of systemic inflammation. There was however no clear pattern of systematic inflammation post-exposure for either SUP or BOP when blood and urine data were considered. There were slight decreases in both fibrinogen and platelet counts at 24 h post-SUP exposure start and 7 h and 24 h post-BOP exposure start. However, a decrease in C-reactive protein (CRP) in the blood (7 h post-SUP exposure start) and in 8-iso-PGF (48 h post-SUP and 24 h/48 h post-BOP exposure start) in urine was also an unexpected finding in this study. However, the decrease in 8-iso-PGF might be part due to the increase in levels following FA exposure. In conclusion, exposure to WS, did not result in consistent increases in any systemic biomarkers.

Riddervold et al [31], Forchhammer et al [32], and Bønløkke et al [33] generated soot/ash-dominated WS that contained a combination of inorganic ash, soot and organics. This WS was generated by a conventional woodstove fuelled by beech wood logs. Participants were exposed to two different WS concentration levels (high and low) for 3 h at rest.

Riddervold et al [31] assessed airway effects of WS exposure in terms of changes in spirometry, FeNO, NAL and EBC for atopic subjects in response. The authors reported a change in conductivity and pH level in the EBC for the higher concentration of WS, which was interpreted as a mild inflammatory response. No other detectable changes were reported in spirometry, FeNO or tested NAL cytokines.

Forchhammer et al [32] investigated markers of systemic inflammation, oxidative stress and changes in microvascular function (MVF) for the same exposure study. The publication reported no significant changes in any of the endpoints after either concentration of WS exposure, including DNA damage, cell adhesion, cytokines or MVF. The authors hypothesized that the lack of effects in this study was due to the low pulmonary deposition of particles from this type of fuel and combustion conditions.

The last publication from this human controlled exposure study (Bønløkke et al [33]) measured heart rate variability (HRV) as well as cytokines, pneumoproteins and factors reflecting coagulation and adhesion. The only significant effect of WS exposure was a decrease in interleukin 6 (IL-6) at 6 h after exposure, similar to findings in Barregard et al [25]. However, this result seems to come from an increase in IL-6 in FA baseline. HRV and other endpoints were not affected by the exposure conditions. Despite the hypothesis presented by the authors, no significant effects were seen between the two WS exposure concentrations in this study.

Unosson et al [34] investigated the effects on the cardiovascular system after WS generated from high temperature incomplete combustion resulting in a WS exposure dominated by soot/ash. WS exposure increased the pulse wave velocity, the augmentation index and pressure as well as an increase in heart rate. There was also a noted decrease in heart rate variability post-exposure. The authors concluded that these results could suggest that exposure to WS has acute effects on cardiovascular health and impact cardiovascular disease.

Muala et al [35] assessed the airway effects in the same human exposure study. Lung function and airway inflammation (FeNO) was not altered by WS exposure, but a range of the factors assessed in blood and bronchoscopy samples were significantly affected. In blood, several cell populations increased after WS exposure, including CD16 + CD56+, CD4 + HLADR+, CD8 + HLADR cells. In the bronchoscopic biopsy samples there was an increase in CD3 + lymphocytes (submucosal and epithelial), CD8 + lymphocytes (epithelium), and mast cells (submucosal). In the BW, there were reductions in macrophages, neutrophils, and lymphocytes, soluble intercellular adhesion molecule (sICAM-1), MPO, matrix metallopeptidase 9 (MMP-9), and LDH. Finally, in the BAL samples there were significant increases in total lymphocyte numbers and total glutathione.

Hunter et al [36], uses the same WS exposure set-up as Unosson et al [34] to investigate effects of short exposure at high dose of WS on cardiovascular endpoints. Carboxyhaemoglobin (COHb) increased significantly 3 h after WS exposure, while blood pressure or heart rate was not affected by the exposure. The subject’s responses to bradykinin was increased after WS exposure, with an increased forearm blood flow. The authors conclude that the results suggest that acute exposures of this kind may not lead to cardiovascular events often seen in firefighters.

### Synopsis by endpoint

The findings from these 12 studies (across 22 publications) may be better understood by synthesizing the results by endpoint, as summarized in Tables 2,3,4,5, where effects on lung inflammation, circulating cells and proteins, oxidative stress, and cardiovascular physiology are presented, respectively. Lung function (not summarized in table) was investigated by spirometry in 5 studies [16, 20, 23, 31, 35] but no significant changes in principal spirometry values (FEV_1_, FVC, or FEV_1_/FVC) were observed in any of the studies, even though different concentrations and properties of WS were applied.

Effects of WS exposure on airway inflammation was addressed in 9 different publications in a range of biological samples, including FeNO, EBC, and bronchoscopy (Table 2). Most endpoints were assessed in only one or two of the publications, which often differed in WS exposure conditions, making cross-study synthesis difficult. The most commonly assessed endpoints between these publications were FeNO (5 studies) and neutrophil count in BAL/BW samples (3 studies). However, none of these endpoints showed an entirely consistent signal across publications. FeNO 270, considered to be a marker of distal NO, was increased at multiple timepoints across two publications using similar WS combustion conditions [26, 29], with null results in three others. Cytokines and cell recruitment also showed inconsistent signals across studies with either significant or null findings, while EBC pH and bronchial wash neutrophils showed conflicting results. Neutrophil numbers were decreased at 24 h in Muala et al [35], but increased at 20 h in Ghio et al [16]. As these studies used very different WS exposures, dominated by either organics or soot/ash, differences in WS exposures and PM classes could possibly contribute to this inconsistency. Notably, the latter finding is supported by the increased sputum percentage neutrophils (%PMNs), a close approximation of BW, in Burbank et al [17] using similar WS combustion conditions.

Markers of circulating cells and proteins were assessed in 10 publications (Table 3) in samples from blood and urine. The most common markers examined were platelets, leukocytes, IL-6, vWf, tumor necrosis factor alpha (TNF-α) and CC16 in blood and CC16 in urine. Again, relatively few studies (between two and four) assessed each endpoint and there were many null findings and several inconsistencies. For several endpoints, two to four studies consistently reported null findings, including leukocytes, red blood cell (RBC), surfactant protein A (SP-A), soluble platelet selectin (sP-selectin), vWf and TNF-α (Table 3). Only two markers showed consistent results across the different studies: CC16 was increased in blood in two studies, while IL-6 was decreased (although in [33] this change could be due to an increase in IL-6 at baseline during FA exposure). The latter was contrary to expectation as it is amongst the markers most consistently elevated in terms of traffic-related air pollution. Notably, the IL-6 level was also significantly decreased in NAL, but not affected in bronchial samples (Table 2).

Twelve publications assessed different markers of oxidative stress, most extensively looking at BAL/BW antioxidant markers (such as GSH, oxidized glutathione (GSSG), and total glutathione (GSx)) and a range of markers in blood, while EBC and urine oxidative stress markers were narrowly evaluated (Table 4). Each endpoint was only assessed in 1–2 studies and there were many null findings and some inconsistencies. In some cases, findings within the same study were also inconsistent [24]. One of the most studied oxidative stress signals in general, 8-isoprostane in urine, was only assessed in two publications, which showed opposite results (although as mentioned previously, the decrease seen in Stockfelt et al [30] in likely due to in part and increase following the control exposure).

Effects of WS exposure on cardiovascular physiology was investigated in seven publications, looking most commonly at heart rate variability and electrocardiogram (ECG) readings (Table 5). Most endpoints were included in two to four of the publications, but there was little consistency. Forchhammer et al [32] showed no change in microvascular function (post-ischemia peripheral artery tonometry). Similarly, in Pope et al [21] the controlled exposure did not alter vascular response (here too assessed by reactive tonometry). However, Unosson et al [34] was noteworthy in demonstrating both increased arterial stiffness and decreased heart rate variability in key metrics, which are considered adverse reactions. Ghio et al [16], however, showed a decrease in maximal heart rate but only a marginal (non-significant) change in high frequency component of HRV (Bønløkke et al. [33] showed no change in HRV). These discrepant findings regarding HRV are perhaps due to exposure time (Unosson et al [34] longer than Ghio et al) and/or inclusion of exercise (which Bønløkke et al [33] did not). Hunter et al [36] did not reproduce Ghio et al [16] maximal heart rate findings upon applying the same exposure setup for WS exposures but using a third of the exposure time and a three-fold higher concentration. This discrepancy could be due to the differences in WS exposure or due to the differences in participant demographics (i.e. healthy non-smokers vs. healthy firefighters). Instead in Hunter et al [36], a significant increase in the response to bradykinin was observed. Fedak et al [19] demonstrated an increase in systolic blood pressure with use of wood-burning cookstoves, in contrast to two of the wood stove studies [34, 36].

Overall, there was most consistency in reported effects for airways (FeNO, neutrophils, CC16), while oxidative stress, systemic inflammation and cardiovascular physiology did not show any clear patterns. However, the large differences in study design, assessed endpoints, sampling times, and WS exposure conditions (concentration, exercise intensity, PM classes) make it difficult to draw conclusions regarding (i) consistent responses within the four classes of endpoints, or (ii) the importance of physicochemical properties of WS PM.

## Discussion

Perhaps the most important observation from this review is that there is great heterogeneity across study designs, in terms of stove, fuel, exposure duration, exercise during exposure, as well as selection of endpoints and timing. Accordingly, it is difficult to know whether the lack of a clear signal in these data is primarily due to heterogenous study designs or to a *bonafide* lack of clinically relevant pathophysiology from WS especially in the acute setting. This makes it difficult to choose a particular signal upon which to focus subsequent efforts. Such efforts are likely to remain dictated in large part by the type of physiological derangement of primary interest by the investigators, though some guidance is provided by the results summarized here.

The current data also suggest that future studies should pay particular attention to the details of the WS exposure (effective dose, clearance rate, and inherent differences a given WS’ toxic potential). In addition, the applied exposure conditions should reflect the research question of the study, as both exposure levels and PM properties vary considerably between wildfires, indoor household emissions in developing countries and outdoor residential wood combustion in developed countries.

As several studies report significant effects of various wood smoke exposures on both respiratory and cardiovascular endpoints [16, 26, 34], one should certainly not conclude that inhalation of WS is benign. Also, these studies assessed acute scenarios only, and generally focused on healthy adults who are likely resilient to such insults. Furthermore, some anatomic compartments (such as the upper airway) were minimally examined or by only very limited endpoints. Even in the lower airway, only 3 studies directly examined the lungs (by bronchoscopy) and so this represents a truly scarce set of evidence obtained precisely from the compartment that would, arguably, be most affected by WS. Similarly, regarding cardiovascular endpoints, only 2 studies investigated arterial stiffness and only 1 study investigated forearm blood flood. As a final example of minimal coverage of key endpoints, we note that only 2 studies investigated oxidative damage to DNA. There was an absence of attention to the role of co-exposures [41], systemic cellular immune responses (and related genetic predispositions) [42] or anti-microbial defence [43], important within the broader air pollution literature; these gaps should be addressed in future studies.

In design of future controlled WS exposure studies we emphasize the importance of including endpoints that have shown the most consistency, especially those that are simple to perform, such as FeNO. In addition, methods that have been of particular insight in controlled human studies of other particulate-rich pollutants, should be prioritized, such as endobronchial brushing and biopsy that were underutilized in the reviewed WS studies. With regard to cardiovascular endpoints, we consider arterial stiffness and heart rate variability as key metrics for future studies, as these are sensitive biomarkers. Moreover, harmonizing of time-points would allow for more interstudy comparison; 24 h post-exposure is a preferred option given its ability to minimize diurnal variability, but the timing should be tightly aligned with known biological responses (some of which peak and fade before 24 h). Finally, multicentre studies with identical (or near-identical) protocols should be pursued to increase consistency and statistical power, and also for comparison of WS dominated by different PM classes.

## Conclusion

Although no clear pattern emerges from the reviewed controlled human exposure studies, one cannot conclude that WS is necessarily less toxic than traffic-related air pollution. The latter has had a much longer period of investigation, and many more studies, allowing for a refinement of the methods that has not yet occurred in WS studies. Accordingly, conclusions regarding effects of acute WS exposure on human health are premature and more carefully conducted human studies are needed.

## Supplementary information

**Additional file 1: Table S1.** Overview of the data used as a basis for the categorization of the exposure conditions applied in the 12 human exposure studies in terms of the PM class dominating the exposure. The table lists references (only by name and year to avoid confusion with manuscript reference numbers), and then in bold and underlined the dominating PM class, as OC, soot or ash, or a combination of these PM classes. The categorization is based on the data reported in each study or inferred based on provided information and literature. The stove and fuel type applied in each study is listed, as well as the PM characterization data. In addition, data provided in supporting papers to draw a conclusion with regard to the PM composition is listed.

## Data Availability

All data reviewed and described is either included in this manuscript or available online in the relevant publications.

## Abbreviations

BAL: Bronchoalveolar lavage
BOP: Burn-out phase
BW: Bronchial wash
CC16: Club cell protein-16
COHb: Carboxyhaemoglobin
CRP: C-reactive protein
EBC: Exhaled breath condensate
EC: Elemental carbon
ECG: Electrocardiogram
FA: Filtered air
FEV1: Forced expiratory volume
FVC: Forced vital capacity
FeNO: Fractional exhaled nitric oxide
GSH: Glutathione
GSSG: Oxidized glutathione
GSTM1: Glutathione S-transferases M1
GSx: Total glutathione
hHO1: Histone H1
hOGG1: Human 8-Oxoguanine glycosylase
hNUDT1: Human MutT homolog 1
HRV: Heart rate variability
IL-1β: Interleukin-1beta
IL-6: Interleukin-6
IP-10: Interferon gamma-induced protein 10
LDH: Lactate dehydrogenase
LOOH: Lipid hydroperoxides
MDA: Malondialdehyde
MMP-9: Matrix metallopeptidase-9
MPO: Myeloperoxidase
MVF/MVRI: Microvascular responsiveness index/Microvascular function
NAL: Nasal lavage
NO: Nitric oxide
OC: Organic carbon
PAHs: Polycyclic aromatic hydrocarbons
PM: Particulate matter
PM2.5: Particulate matter of less than or equal to 2.5 μm in aerodynamic diameter
pNN50: Percentage of successive NN interval greater than 50 ms
RBC: Red blood cell
RMSSD: Square root of the mean squared differences of successive NN intervals
SAA: Serum Amyloid A
SDNN: Standard deviation of the NN interval
sICAM-1: Soluble intercellular adhesion molecule-1
SP-A: Surfactant protein A
SP-D: Surfactant protein D
sP-selectin: Soluble platelet selectin
SUP: Start-up phase
sVCAM-1: Soluble vascular cell adhesion molecule-1
TEAC: Trolox equivalent antioxidant capacity
TNF-α: Tumor necrosis factor alpha
UA: Uric acid
VIIIc: Factor VIII complex
WS: Wood smoke
vWf: Von Willebrand factor
3-NT: 3-nitrotyrosine
8-iso-PGF2: 8-Iso-prostaglandin F2alpha
8-iso: 8-Isoprostane
8-oxoGua: 8-Oxo-7,8-dihydroguanine
8-oxodG: 8-Oxo-2′-deoxyguanosine
%PMN: Percentage sputum neutrophils
