# Supplementary material for: Controlled human exposures to wood smoke: a synthesis of the evidence
Source: Part Fibre Toxicol. 2020 Oct 2;17:49. doi: 10.1186/s12989-020-00375-x (PMC7530963; doi:10.1186/s12989-020-00375-x)
Supplement: Supplementary file 1 — Additional file 1: Table S1. Overview of the data used as a basis for the categorization of the exposure conditions applied in the 12 human exposure studies in terms of the PM class dominating the exposure. The table lists references (only by name and year to avoid confusion with manuscript reference numbers), and then in bold and underlined the dominating PM class, as OC, soot or ash, or a combination of these PM classes. The categorization is based on the data reported in each study or inferred based on provided information and literature. The stove and fuel type applied in each study is listed, as well as the PM characterization data. In addition, data provided in supporting papers to draw a conclusion with regard to the PM composition is listed. [file 12989_2020_375_MOESM1_ESM.docx]

# **Supplementary material**

The basis for the categorization of the exposure conditions applied in the 10 human exposure studies, in terms of the PM class dominating the exposure, is provided here (Table S1), with additional rationale for why a given study exposure is designated as dominated by inorganic ash, soot, or organic carbon (OC), or a combination of these PM classes. For the American studies, the physicochemical properties of the PM applied is characterized based on information provided regarding stove type and fuel, in combination with available literature regarding how such factors influence wood smoke PM characteristics (see table S1 for details). The studies are ordered based on their combustion conditions and dominating PM classes, starting with smouldering (OC dominated), followed by low temperature flaming combustion (OC/soot dominated) and more complete combustion conditions (soot/inorganic ash).

The three studies using heating elements [1-3] did not provide any information regarding the physicochemical PM properties of the exposure. The authors do not provide the exact model of the heating element, but it appears that an element without temperature control was applied, most likely resulting in smouldering combustion. Kim et al. [4] applied a similar smoke generation system, with temperature control, to generate both smouldering and flaming combustion conditions. A detailed chemical analysis of the generated PM suggests that the method provides exposures dominated by OC and to a certain extent inorganic ash. Virtually no elemental carbon was detected. The fraction of inorganic elements and ionic species indicative of alkali salts ranged from about 0.15 to 0.40 for the flaming combustion condition (depending on wood species), but was very low (< 0.03) for smouldering conditions. These data suggest that PM generated using the electric heating element are dominated by OC and possibly inorganic ash, depending applied combustion conditions and wood species. Since the heating element applied in the human exposure studies appeared to be without temperature control, we suggest that human smoke exposures in these studies were dominated by OC [1-3].

**Table S1:** Overview of the data used as a basis for the categorization of the exposure conditions applied in the 12 human exposure studies in terms of the PM class dominating the exposure. The table lists references (only by name and year to avoid confusion with manuscript reference numbers), and then in bold and underlined the dominating PM class, as OC, soot or ash, or a combination of these PM classes. The categorization is based on the data reported in each study or inferred based on provided information and literature. The stove and fuel type applied in each study is listed, as well as the PM characterization data. In addition, data provided in supporting papers to draw a conclusion with regard to the PM composition is listed.

| Ghio et al. (2012)[1] | **OC dominated**  Data provided in publication:   - Description of heating element and fuel conditions - Measured: PM mass, PM number and number size distribution - No EC/OC/inorganic ash measurements or other description of PM properties   Supplementary information:  Kim et al 2018 applied a similar smoke generation system, and applied both smoldering and flaming combustion conditions. A detailed chemical analysis of the generated PM suggests that the method provides exposures dominated by OC and to a certain extent inorganic ash. Virtually no elemental carbon was detected. The fraction of inorganic elements and ionic species indicative of alkali salts ranged from about 0.15 to 0.40 for the flaming combustion condition depending on wood species, but was very low for smouldering conditions (< 0.03). These data suggest that PM generated using the electric heating element appear to be dominated by OC and possibly inorganic ash, depending applied combustion conditions and wood species.  Since smoldering combustion seems to be applied in Ghio et al. [1], **OC is likely to be dominating PM types** due to application of heating element. |
| --- | --- |
| Burbank et al. (2019) | **OC dominated**  Data provided in publication:   - as Ghio et al. [1]   Since conditions are similar as Ghio et al. [1], **OC is likely to be dominating PM types** due to application of heating element. |
| Rebuli et al. (2019) | **OC dominated**  Data provided in publication:   - Description of heating element and fuel conditions - Measured: PM mass, PM number and number size distribution, CO, O3, NOx, SO2, total hydrocarbon - No EC/OC/inorganic ash measurements or other description of PM properties   Since smoldering combustion seems to be applied in Rebuli et al. [3]., **OC is likely to be dominating PM types** due to application of heating element (see Ghio et al. [1] for supporting data). |
| Fedak et al. (2019) | **OC or soot dominated (comparing different stoves)**  Five different stoves/atmospheres were compared, one for propane, one for wood chips (gasifier) and three for wood sticks (fan rocket, rocket elbow and three stone fire)  Data provided in publication:   - Description of heating element and fuel type (no data on wood species or moisture content) - Measured: PM_2,5_ mass, PM number and number size distribution, EC, OC, EC/PM_2,5_ ratio, NO, NO2, selected total hydrocarbons - No inorganic ash measurements - The EC and OC percentages of total carbon (TC) provide information about the dominating PM species for the four wood fired stoves   - **Gasifier**: OC ~ 22 %, EC ~ 78% **→ EC dominated**   - **Fan rocket**: OC ~ 32 %, EC ~ 68% **→ EC dominated**   - **Rocket elbow**: OC ~ 31 %, EC ~ 69% **→ EC dominated**   - **Three stone fire**: OC ~ 81 %, EC ~ 19% **→ OC dominated**   Based on the provided OC/EC data, **soot or OC are likely to be dominating particle types, depending on the stove and fuel type.** |
| Sehlstedt et al. (2010) | **OC dominated (with soot and inorganic ash); OC/soot**  Data provided in publication:   - Pellet stove/ Fuel: pellets/saw dust - Measured:1,3-butadiene, benzene, PM2,5, PM mass size distribution (aerodynamic diameter, equivalent mobility diameter, OC/EC, major inorganic ions, PHAs)   The authors indicated the fractions of the three PM classes to be **25%EC, 60% OC, 13% alkali salts.** Since OC and soot were the two PM classes with highest reported percentages, we classified the exposure as **OC/soot dominated**. |
| Pope et al. (2011) | **OC dominated (with soot and inorganic ash); OC/soot**  Data provided in publication and supporting paper (Kuprov et al 2011):   - Conventional stove/logs - Exposure system included UV ageing system, generating ages wood smoke PM - Measured: PM mass, PM chemistry (refractory elements, anions). EC and OC content, as well as gasses (CO, CO_2_, NO_x_, O_3_) - Results: Chemical analysis of elements: sum of refractory elements/total mass fraction ~ 10-12% (indicative of alkali salts/inorganic ash). Elemental carbon 23 +/- 12%, organic matter 68 +/- 12 %.   Based on provided chemical analysis data **OC** and soot **are likely to be dominating particle types.** Since OC and soot were the two PM classes with highest reported percentages, we classified the exposure as **OC/soot dominated**. |
| Ferguson et al. (2016) | **OC/soot dominated**  Data provided in publications:   - Conventional stove/logs - Description of stove type and fuel conditions. Data for PM2,5, CO, CO2 - No EC/OC/inorganic ash measurements or other description of PM properties   Based on information regarding stove and fuel type, and comparison with Nordic studies using similar stoves and fuel, it appears that **elemental carbon and OC are likely to be dominating particle types** |
| Ferguson et al. (2017) |  |
| Peters et al. (2018) |  |
| Barregard et al. (2006) | **OC/soot dominated**  Data provided in publications and supporting publication [12]:   - Conventional stove/logs - Measurements of PM2,5, CO, PM0,1, PM0,16, particle number concentration, VOCs, PAHS, element mass concentration (indicative of alkali salts), black smoke (BC) - Results: Chemical analysis of elements: sum of elemental/total mass fraction ~ 5-6% (indicative of alkali salts/inorganic ash). Black smoke 20-50% (indicative of soot)   Based on provided chemical analysis data, soot appears to account for up to 50% of the PM, while inorganic ash seems to have a relatively small contribution to the total PM mass. Thus, OC must also be a dominating PM class. In conclusion, the provided data suggest that OC **and soot are likely to be dominating particle types.** |
| Barregard et al. (2008) |  |
| Danielsen et al. (2008) |  |
| Murgia et al. (2016) |  |
| Stockfelt et al. (2012) | **OC/soot dominated**  Data provided in publications:   - Conventional stove/logs - Same setup and combustion conditions as the Barregard/Sällsten study, but different campaign.   Since similar combustion conditions were applied as in the Barregard/Sällsten study OC and **soot are likely to be dominating particle types.** |
| Stockfelt at al. (2013) |  |
| Riddervold et al. (2011)  Riddervold et al. (2012) | **Soot/inorganic ash dominated**  Data provided in publications:   - Applied 2-stage stove (more complete combustion conditions than conventional wood stove) - Measured: TSP, PM_2.5_, PM_10_, particle number distribution, and in supporting paper also levels of levoglucosan and a selection of 8 PAHs - In Riddervold et al. 2011 a bimodal size distribution is reported. The count mean diameter of the first peak is located at about Dp = 50–60 nm and can probably be allocated to soot particles while the second peak is located at about Dp = 140–160 nm and is most likely composed of a combination of organic matter and inorganic salts. The authors suggest that smaller particles may be composed to a larger extent by alkali salts and the larger ones by black carbon. As organic matter in this combustion process enters the particle phase by condensation, it might be distributed over the full-size range with varying mass ratios. - No mass fraction indicated, but supporting paper [15] suggests that the PM **is most likely a combination of alkali salts (inorganic ash), black carbon (soot) and organic matter (OC)**   Since the stove is a two-stage stove, providing more complete combustion conditions, and the authors suggest two peaks in size distribution dominated by either soot or inorganic ash, **soot and inorganic ash are likely to be dominating PM types.** |
| Forchammer et al. (2012) |  |
| Bønløkke et al. (2014) |  |
| Unosson et al. (2013) | **Soot/inorganic ash dominated**  Data provided in publications and supporting papers [23]   - Conventional stove/logs - Measured: PM mass, total particle number, number size distribution, TC, EC, PAHs. - Interpretation of relevance of PM exposure conditions in supporting paper: The emissions from Swedish wood stoves, comparing a Swedish field study, are covered fairly well with the applied methodology, but other field studies report considerably higher emissions especially for diluted particle sampling.   The author indicated the fractions of the three PM classes to be **38% soot, 24% OC, 38% inorganic ash**. |
| Muala et al. (2015) |  |
| Hunter et al. (2014) | **Soot/inorganic ash dominated**  Data provided in publication:   - Same stove/similar combustion conditions as Unosson et al 2013, i.e. described in Pettersson et al 2011   The author indicated the fractions of the three PM classes to be **38% soot, 24% OC 38% inorganic ash**. |

References:

1. Ghio AJ, Soukup JM, Case M, Dailey LA, Richards J, Berntsen J, Devlin RB, Stone S, Rappold A. 2012. Exposure to WS particles produces inflammation in healthy volunteers. Occup Environ Med. 69(3):170-175.
2. Burbank AJ, Vadlamudi A, Mills KH, Alt EM, Wells H, Zhou H, Alexis N, Hernandez ML, Peden DB. 2019. The glutathione-S-transferase mu-1 null genotype increases WS-induced airway inflammation. J Allergy Clin Immunol. 143(6):2299-2302.
3. Rebuli ME, Speen AM, Martin EM, Addo KA, Pawlak EA, Glista-Baker E, Robinette C, Zhou H, Noah TL, Jaspers I. 2019. WS exposure alters human inflammatory responses to viral infection in a sex-specific manner: A randomized, placebo-controlled study. Am J Respir Crit Care Med. 199(8):996-1007.
4. Kim YH, Warren SH, Krantz QT, King C, Jaskot R, Preston WT, George BJ, Hays MD, Landis MS, Higuchi M, DeMarini DM, Gilmour MI. 2018. Mutagenicity and lung toxicity of smoldering vs. flaming emissions from various biomass fuels: implications for health effects from wildland fires. Environ Health Perspect. 126(1): 1-14.
5. Fedak KM, Good N, Walker ES, Balmes J, Brook RD, Clark ML, Cole-Hunter T, Devlin R, L’Orange C, Luckasen G, *et al*. 2019. Acute effects on blood pressure following controlled exposure to cookstove air pollution in the STOVES study. J Am Heart Assoc 8:012246
6. Sehlstedt M, Dove R, Boman C, Pagels J, Swietlicki E, Londahl J, Westerholm R, Bosson J, Barath S, Behndig AF, et al. 2010. Antioxidant airway responses following experimental exposure to WS in man. Part Fibre Toxicol. 7:1-11.
7. Pope AC, Hansen JC, Kuprov R, Sanders MD, Anderson MN, Eatough DJ. 2011. Vascular function and short-term exposure to fine particulate air pollution. J Air Waste Manag Assoc 61:858-63.
8. Ferguson MD, Semmens EO, Dumke C, Quindry JC, Ward TJ. 2016. Measured pulmonary and systemic markers of inflammation and oxidative stress following wildland firefighter simulations. J Occup Environ Med. 58(4):407-413.
9. Ferguson MD, Semmens EO, Weiler E, Domitrovich J, French M, Migliaccio C, Palmer C, Dumke C, Ward T. 2017. Lung function measures following simulated wildland firefighter exposures. J Occup Environ Hyg. 14(9):739-748.
10. Peters B, Ballmann C, Quindry T, Zehner EG, McCroskey J, Ferguson M, Ward T, Dumke C, Quindry JC. 2018. Experimental woodsmoke exposure during exercise and blood oxidative stress. J Occup Environ Med. 60(12):1073-1081.
11. Barregard L, Sällsten G, Gustafson P, Andersson L, Johansson L. 2006. Experimental exposure to wood-smoke particles in healthy humans: effects on markers of inflammation, coagulation, and lipid peroxidation. Inhal Toxicol. 18(11):845-853.
12. Sällsten G, Gustafson P, Johansson L, Johannesson S, Molnar P, Strandberg B, Tullin C, Barregard L. 2006. Experimental WS exposure in humans. Inhal Toxicol. 18(11):855-864.
13. Barregard L, Sällsten G, Andersson L, Almstrand AC, Gustafson P, Andersson M,

Olin AC. 2008. Occup Environ Med. 65(5):319-324.

1. Danielsen PH, Brauner EV, Barregard L, Sällsten G, Wallin M, Olinski R, Rozalski R, Moller P, Loft S. 2008. Oxidatively damaged DNA and its repair after experimental exposure to WS in healthy humans. Mutat Res. 642(1-2):37-42.
2. Murgia N, Barregard L, Sallsten G, Almstrand AC, Montuschi P, Ciabattoni G, Olin AC. 2016. 8-isoprostande in exhaled breath condensate after experimental exposure to wood smoke in humans. J Biol Reg Homeos Ag. 30(1):263-270
3. Stockfelt L, Sällsten G, Olin AC, Almerud P, Samuelsson L, Johannesson S, Molnar P, Strandberg B, Almstrand AC, Bergemalm-Rynell K, Barregard L. 2012. Effects on airways of short-term exposure to two kinds of WS in a chamber study of healthy humans. Inhal Toxicol. 24(1):47-54.
4. Stockfelt L, Sällsten G, Almerud P, Basu S, Barregard L. 2013. Short-term chamber exposure to low doses of two kinds of WS does not induce systemic inflammation, coagulation or oxidative stress in healthy humans. Inhal Toxicol. 25(8):417-425.
5. Riddervold IS, Bønløkke JH, Molhave L, Massling A, Jensen B, Grønborg TK, Bossi R, Forchhammer L, Kjaergaard SK, Sigsgaard T, et al. 2011. WS in a controlled exposure experiment with human volunteers. Inhal Toxicol. 23(5):277-288.
6. Riddervold IS, Bønløkke JH, Olin AC, Gronborg TK, Schlunssen V, Skogstrand K, Hougaard D, Massling A, Sigsgaard T. 2012. Effects of WS particles from wood-burning stoves on the respiratory health of atopic humans. Part Fibre Toxicol. 9:12.
7. Forchhammer L, Moller P, Riddervold IS, Bønløkke J, Massling A, Sigsgaard T, Loft S. 2012. Controlled human WS exposure: oxidative stress, inflammation and microvascular function. Part Fibre Toxicol. 9:1-11.
8. Bønløkke JH, Riddervold IS, Gronborg TK, Skogstrand K, Hougaard DM, Barregard L, Sigsgaard T. 2014. Systemic effects of WS in a short-term experimental exposure study of atopic volunteers. J Occup Environ Med. 56(2):177-183.
9. Unosson J, Blomberg A, Sandstrom T, Muala A, Boman C, Nystrom R, Westerholm R, Mills NL, Newby DE, Langrish JP, Bosson JA. 2013. Exposure to WS increases arterial stiffness and decreases heart rate variability in humans. Part Fibre Toxicol. 10(1):1-8
10. Pettersson E, Boman C, Westerholm R, Boström D, Nordin A: Stove performance and emission characteristics in residential wood log and pellet combustion, part 2: wood stove. Energy Fuel 2011, 25:315–323.
11. Muala A, Rankin G, Sehlstedt M, Unosson J, Bosson JA, Behndig A, Pourazar J, Nystrom R, Pettersson EE, Bergvall C, et al. 2015. Acute exposure to WS from incomplete combustion - indications of cytotoxicity. Part Fibre Toxicol. 12(1):1-14.
12. Hunter AL, Unosson J, Bosson JA, Langrish JP, Pourazar J, Raftis JB, Miller MR, Lucking AJ, Boman C, Nyström R, et al. 2014. Part Fibre Toxicol. 11(1):1-13.

﻿
